# Evaluating Novel SP-B and SP-C Synthetic Analogues for Pulmonary Surfactant Efficacy

**DOI:** 10.7150/ijms.92920

**Published:** 2024-02-25

**Authors:** Chae Young Kim, Sung-Hoon Chung, Yong Sung Choi, Kyeong-Yong Park, Chong-Woo Bae

**Affiliations:** 1 Department of Pediatrics, Kyung Hee University School of Medicine, Kyung Hee University Hospital at Gangdong, Seoul, South Korea.; 2 Department of Pediatrics, Kyung Hee University School of Medicine, Kyung Hee University Hospital, Seoul, South Korea.; 3 Department of Integrated Material's Development, CHA Meditech Co., Ltd, Daejeon, South Korea.; 4 Department of Pediatrics, CHA Ilsan Medical Center, CHA University School of Medicine, Goyang, South Korea.

**Keywords:** pulmonary surfactants, synthetic, pulmonary surfactant-associated protein B, disulfide bond, surface tension

## Abstract

Pulmonary surfactants, a complex assembly of phospholipids and surfactant proteins such as SP-B and SP-C, are critical for maintaining respiratory system functionality by lowering surface tension (ST) and preventing alveolar collapse. Our study introduced five synthetic SP-B peptides and one SP-C peptide, leading to the synthesis of CHAsurf candidates (CHAsurf-1 to CHAsurf-5) for evaluation. We utilized a modified Wilhelmy balance test to assess the surface tension properties of the surfactants, measuring spreading rate, surface adsorption, and ST-area diagrams to comprehensively evaluate their performance. Animal experiments were performed on New Zealand white rabbits to test the efficacy of CHAsurf-4B, a variant chosen for its economic viability and promising ST reduction properties, comparable to Curosurf®. The study confirmed that higher doses of SP-B in CHAsurf-4 are associated with improved ST reduction. However, due to cost constraints, CHAsurf-4B was selected for in vivo assessment. The animal model revealed that CHAsurf-4B could restore alveolar structure and improve lung elasticity, akin to Curosurf®. Our research highlights the significance of cysteine residues and disulfide bonds in the structural integrity and function of synthetic SP-B analogues, offering a foundation for future surfactant therapy in respiratory disorders. This study's findings support the potential of CHAsurf-4B as a therapeutic agent, meriting further investigation to solidify its role in clinical applications.

## Introduction

Pulmonary surfactant plays a critical role in maintaining the physiological function of the respiratory system by reducing the surface tension (ST) at the air-liquid interface within the alveoli 1. It primarily consists of phospholipids and surfactant proteins (SPs), that contribute to the stabilization and elasticity of the alveolar membranes 2. Among these, SP-B significantly enhances respiratory function by facilitating the optimal distribution of surfactant along the alveolar surface, and in synergy, SP-C augments these effects by integrating into the surfactant's lipid layers, thereby improving the longevity of the lipid film and preserving alveolar structure integrity 3, 4. In our previous study 5, we addressed the challenges related to composition and functionality as well as ST dynamics by developing a synthetic surfactant that included appropriate lipids and SPs. We developed a synthetic surfactant that includes a mimic of SP-B, along with a carefully formulated composition of appropriate lipids and SPs.

The inspiration to modify the SP-B sequence stemmed from the promising results demonstrated by advanced SP-B peptide mimics, such as Mini-B, Super Mini-B, or B-YL surfactant 6, 7. However, despite their efficacy, the complexity of synthesizing these peptides posed challenges. The focus thus shifted towards developing a peptide that maintained the essential functions of reducing surface tension at the air-liquid interface and preserving alveolar structures, yet could be synthesized more efficiently. Building on our previous research 5, 8, refined the amino acid sequence of a SP-B mimic to enhance its bioactivity and stability while simplifying its production for broader clinical application. In our selection of the SP-C analogue, SP-CL16 (6-28) was chosen despite its absence of the N-terminus and palmitoylation, characteristics of human SP-C, based on its effectiveness in prior research 9. This decision reflects our strategy to balance synthesis practicality with the efficacy required for our synthetic surfactant's development, even against the backdrop of more complex but potentially more effective sequences like SP-C33 10. The use of SP-CL16 (6-28) capitalizes on its ability to enhance hydrophobic interactions within the lipid bilayer, crucial for the surfactant's performance 11.

Through careful design and development, the goal was to achieve an optimal balance between biocompatibility, stability, and surface activity of the SP-B mimic. In pursuing this goal, we meticulously designed and adjusted the amino acid sequence, essential for effective surfactant replacement in respiratory distress syndromes, identifying modifications that could enhance performance without compromising functionality.

## Materials and Methods

### Synthetic pulmonary surfactant preparation

In this study, the design of synthetic pulmonary surfactants began with the adoption of the SP-B peptide sequence (RMLPQLVCRLVLRCSMD) from prior research as a foundational structure for modification 5, 8. The structural modifications to this peptide were meticulously chosen to enhance its functional properties. Specifically, Methionine (M) was substituted with Norleucine (Nle) to mitigate the risk of oxidation, a prevalent issue that can compromise peptide stability and functionality. This substitution aimed to increase the peptide's resilience in oxidative environments. Similarly, the replacement of Cysteine (C) with Serine (S) was intended to prevent the formation of unintended disulfide bonds that could adversely affect the peptide's correct folding and activity. The alteration from Arginine (R) at the sequence's onset to Cysteine (C) targeted the facilitation of head-to-tail cyclization, which is crucial for the peptide's structural integrity and the promotion of intra- and inter-molecular disulfide bonding, essential for its surfactant functionality. Moreover, the second Methionine (M) was replaced with Tryptophan (W) to bolster the peptide's amphipathic character, vital for its interactions with lipid components and surfactant efficacy. These strategic modifications resulted in the conception of five distinct SP-B candidates, as detailed in Table 1. For the SP-C analogue, the sequence used was CPVHLKRLLLLLLLLLLLLLLLL, ensuring consistency with the proven research findings. These peptides were synthesized and amalgamated with lipid components in specified ratios to formulate the synthetic surfactants CHAsurf-1 through CHAsurf-5, alongside additional variants CHAsurf-4A, 4B, 4C, and 4D developed following initial experiments, as shown in Table 2. The synthesis involved dissolving the peptides along with DPPC, POPG, and PA in trifluoroacetic acid, followed by a mixture with CHCl3 and CH3OH. A 10% ethanol solution was added before the evaporation process at 40-45°C for 15 minutes, culminating in solid components ready for lyophilization.

### *In vitro* experiments

In the previous study conducted at Iwate Medical School in Japan, the modified Wilhelmy balance machine was utilized to measure ST. In light of travel restrictions due to the COVID-19 pandemic, we constructed a new modified Wilhelmy balance machine, as access to the one at Iwate Medical School in Japan was not feasible. Our apparatus included a motor-operated, plunger-equipped Teflon water tank (15.0 × 6.0 × 2.2 cm) connected to the K20 tensiometer (Krüss, Germany). We measured ST and maintained a consistent temperature beneath the plunger with a heat plate. For surfactant spreading rate analysis, 1.3 µl of a 25 mg/mL surfactant solution was added to 80 mL of saline in the tank, maintained at 37°C. During the 3.5-minutes observation period, close attention was given to monitoring the spreading behavior of the surfactant on the saline surface. To assess the surfactant-saline interaction, we conducted 66% compression-decompression cycles and specifically focused on the data from the seventh cycle for its stability, from which the ST-area diagram was obtained. For the surface adsorption rate measurement, a circular Teflon tank with 7 mL saline and 1 mL surfactant (0.5 mg/mL) was used, stirred at 120 rpm with a Magnetic Striders MIX 1 echo rotator, and observed for 40 minutes. These methods enabled comparison of our five synthetic surfactant candidates with the commercial product Poractant alfa (Curosurf®, Chiesi Farmaceutici, Parma, Italy). After selecting the most promising candidate, we further analyzed its physical properties by varying SP-B dosages.

### Animal protocol

All experimental procedures received approval from the Kyung Hee University Medical Center's Animal Research and Ethics Committee (KHMC-IACUC 22-042). Animal experiments were conducted using a New Zealand white rabbit model to assess a single promising material's efficacy in respiratory care. Caesarean sections were performed on anesthetized mother rabbits, using 15 mg/kg Zoletil® (tiletamine + zolazepam) administered via the auricular vein, resulting in the extraction of preterm pups on G27 and term pups on G31. Post-delivery, tracheostomies were performed, followed by gentle forced ventilation using a syringe with 0.3-0.5 cc of air, while positioning the fetus in a supine position, cutting a side hole in the trachea, inserting an 18-gauge angiocatheter into the trachea, and positioning the tip of the angiocatheter above the carina using an elastic thread. The study included four groups: 1) preterm control, 2) term control, 3) preterm pups treated with Curosurf®, and 4) preterm pups treated with CHAsurf.

The dosage of the administered surfactant was 100 mg/kg for all treatment groups. Pressure-volume curves during the deflation phase were obtained for all four groups using a small animal ventilator (VentElite, Harvard Apparatus; Cambridge, MA, USA), with the measured volume normalized to body weight. Additionally, pathological findings were analyzed. After the pressure-volume curve test, the tracheostomy was blocked at 10 cm H_2_O and the lung tissue samples were fixed in a formalin solution. H-E staining was performed on both left and right lung tissue samples to observe histologic findings, and the aerated area ratio was analyzed at ×100 magnification using the ImageJ program (V 1.48 for Windows, NIH, Bethesda, MD, USA).

### Statistical analysis

The Mann-Whitney U test was used to compare both lung tissue aeration and lung volumes at a pressure of 25 cm H2O, with a p-value of <0.05 indicating statistical significance. All analyses were conducted with R software (Version 4.2.0, The R Foundation for Statistical Computing, Vienna, Austria).

## Results

### Performance assessment of synthetic surfactants

**Figure 1:**
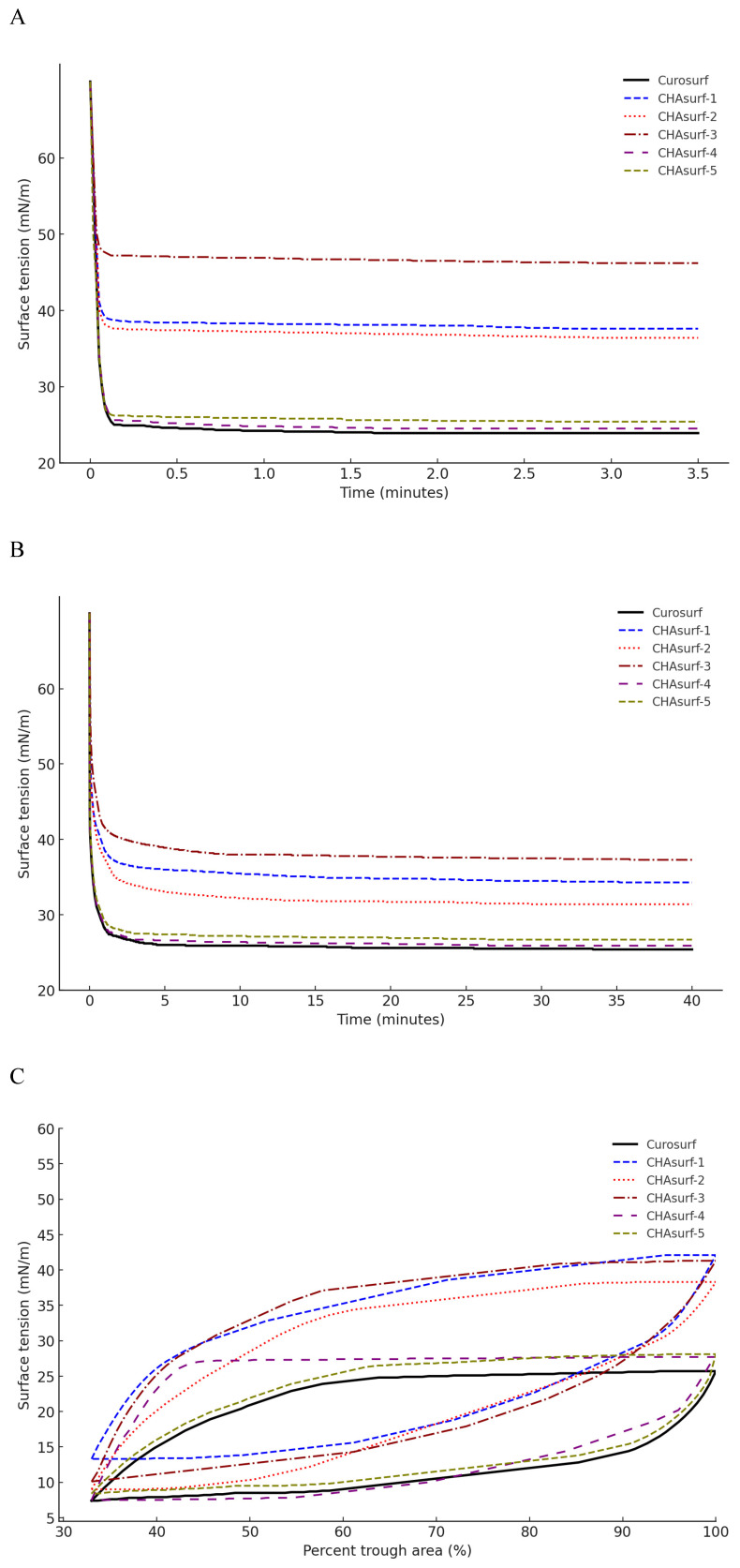
** ST analysis in CHAsurf and Curosurf®: comparing spreading rate, adsorption rate, and ST area diagram.** The modified Wilhelmy balance test (A, B) illustrates that CHAsurf-1, 2, and 3 had slower spreading, higher adsorption rates, and elevated minimum ST. In contrast, CHAsurf-4, 5, and Curosurf® exhibited rapid surface spreading and adsorption rates. The surface tension area diagram (C) revealed hysteresis curves for all five preparations. CHAsurf-4, 5, and Curosurf® shared similar minimum ST values, while CHAsurf-1, 2, and 3 yielded less favorable results. ST, surface tension.

**Figure 2:**
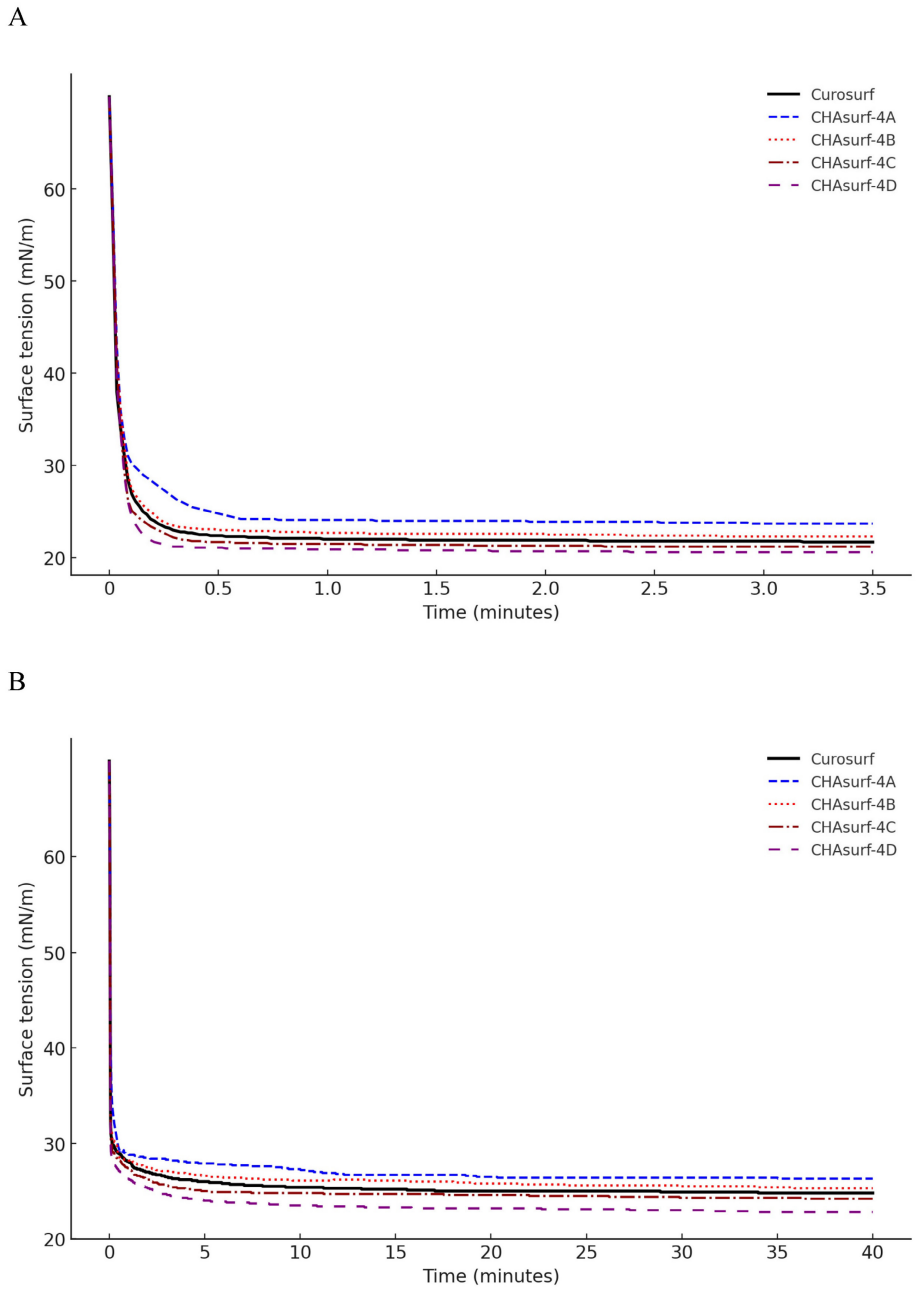

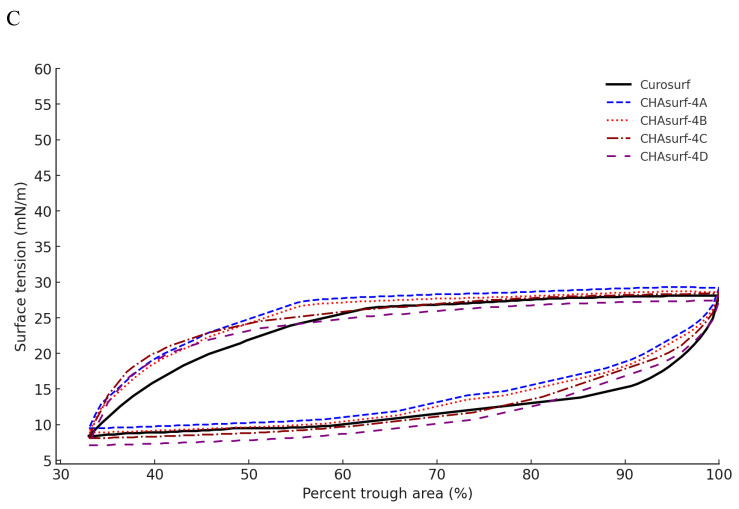
**Impact of SP-B dosage on ST in CHAsurf Formulations and Curosurf®.** The modified Wilhelmy balance test, with diagram (A) focusing on the spreading rate test and diagram (B) on the adsorption rate test, reveals the impact of SP-B content. CHAsurf-4D, containing the highest SP-B dose, demonstrated the fastest spreading and higher adsorption rates. In contrast, CHAsurf-4A, with the lowest SP-B dose, exhibited the slowest performance. CHAsurf-4B and 4C closely matched Curosurf® in performance. Diagram (C), in the ST area, illustrates that lower minimum ST values correspond to the SP-B dosage, with Curosurf® positioned between CHAsurf-4B and 4C. ST, surface tension.

In our study, we conducted a modified Wilhelmy balance test to evaluate five synthetic surfactant candidates, CHAsurf-1 to CHAsurf-5, alongside the commercial product Curosurf®. The surface spreading rate test (Fig. 1A) revealed that CHAsurf-4 and 5, along with Curosurf®, demonstrated notably rapid spreading rates, which contrasted with the slower rates observed in CHAsurf-1 to 3. In the surface adsorption test (Fig. 1B), where Curosurf® led in adsorption efficiency, closely followed by CHAsurf-4 and 5. This was further supported by the ST-area diagram analysis (Fig. 1C), each of the six formulations exhibited distinct hysteresis curves, with CHAsurf-4 and 5, and Curosurf® showing lower minimum ST values, suggesting superior efficiency in surface tension reduction. Based on these findings, we repeated the modified Wilhelmy balance test, narrowing our focus to CHAsurf-4, 5, and Curosurf® for a more detailed comparison. Our additional analysis involved labeling each candidate with A, B, C, and D, corresponding to increasing SP-B dosages (2mg, 3mg, 4mg, and 5mg, respectively), where CHAsurf-4, particularly at higher SP-B dosages, showed a tendency to outperform CHAsurf-5 in reducing ST (Fig. 2).

### Comparative analysis in animal models

Given the demonstrated efficacy of higher SP-B doses in reducing ST, but also considering the cost implications of the peptide, our animal study was specifically focused on CHAsurf-4B. This choice balanced between effectiveness and economic viability. The study utilized 31 preterm and 10 term pups from 8 mother rabbits, with an average birth weight of 36.1±3.1 g for preterm and 64.3±7.6 g for term pups. The experimental groups included 10 term pups for the term control group, 11 preterm pups for the preterm control group, and 10 preterm pups each assigned to the Curosurf® and CHAsurf-4B treatment groups (Table 3).

**Figure 3:**
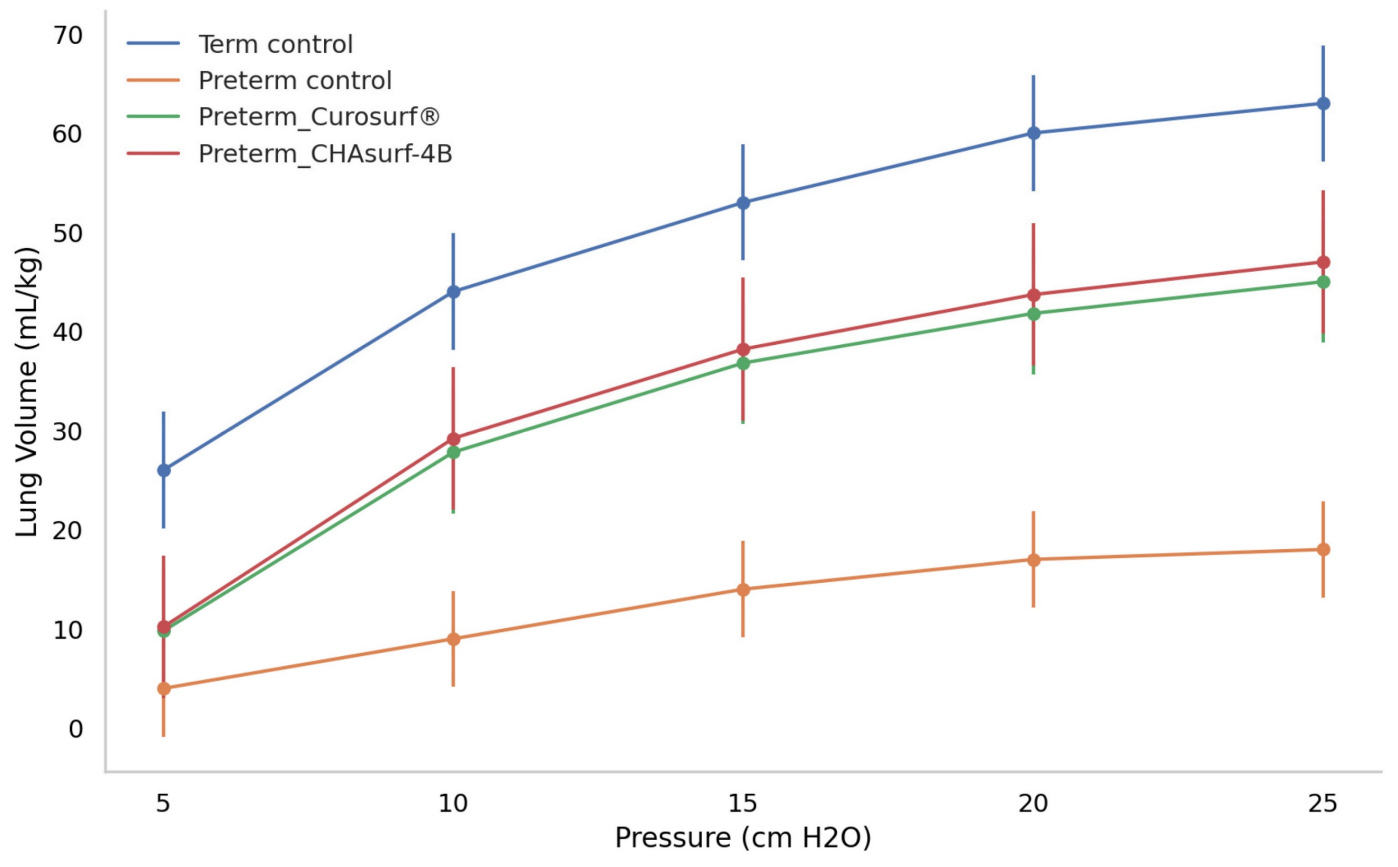
** Deflation curves in term and preterm pups: a comparative analysis of pressure response.** The figure depicts how term control, preterm control, and preterm groups treated with Curosurf® and CHAsurf-4B respond differently under varying pressures. Notably, the term control, Curosurf® (both n=10, p<0.001), and CHAsurf-4B (n=10, p<0.001) groups show higher pressure-volume curves than the preterm control (n=11). The Mann-Whitney U test was applied to evaluate lung volume differences at 25 cm H2O pressure. Vertical lines on the graph indicate the standard deviation.

**Figure 4:**
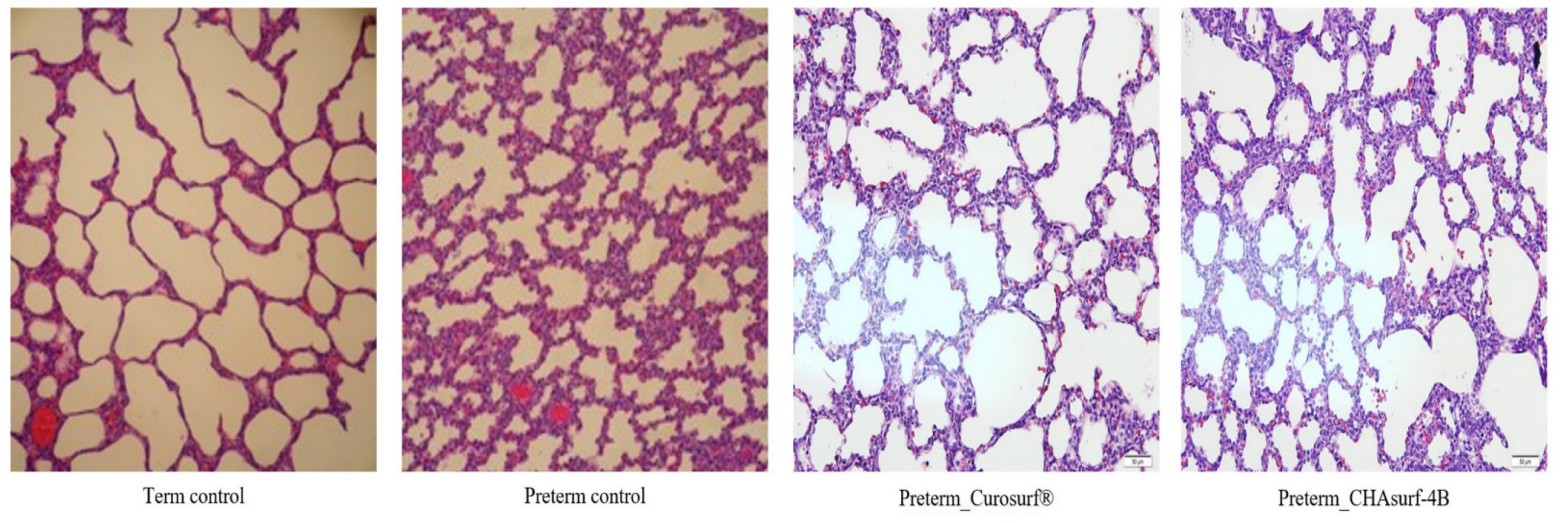
** Comparative histological profiles in four distinct groups (H&E stain, magnification ×200).** Lung tissues treated with CHAsurf-4B and Curosurf® exhibited enhanced aeration characteristics compared to those of preterm controls, although not as pronounced as in the term control samples.

**Figure 5:**
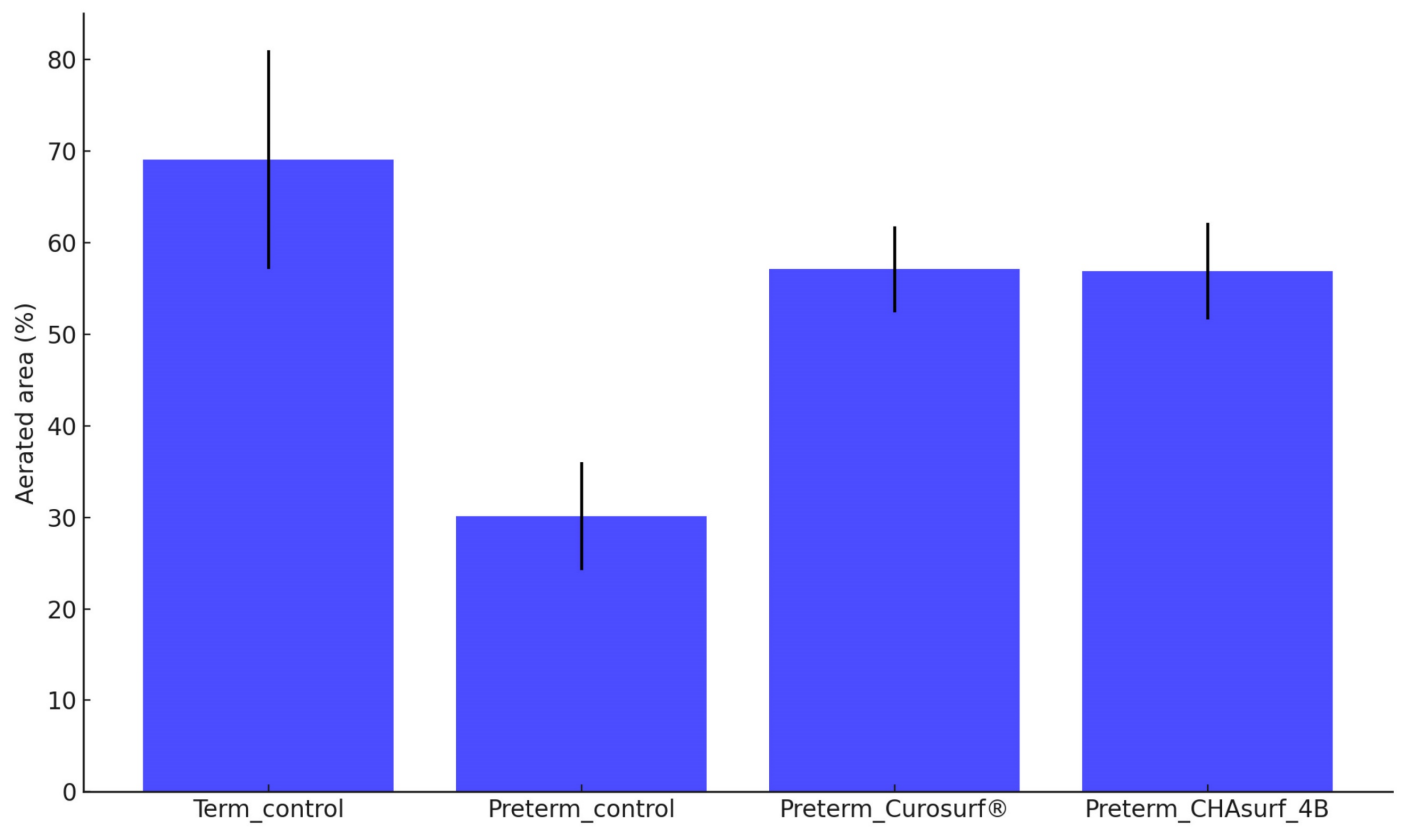
** Aerated area percent comparison.** The bar graph presents the average percentages of aerated areas in lung tissues for four different groups: term control (n=10), preterm control (n=11), preterm_Curosurf® (n=10), and preterm_CHAsurf-4B (n=10). When compared to the Preterm control, tissues treated with Curosurf® and CHAsurf-4B demonstrate a statistically significant increase in aeration, as determined by the Mann-Whitney U test with p-values <0.001 for both treatments. This suggests that the application of Curosurf® and CHAsurf-4B is associated with enhanced aeration in preterm lung tissues. Vertical lines on the graph indicate the standard deviation.

The pressure-volume curve analysis, emphasizing the deflation phase (Fig. 3), revealed distinct lung functionality among the groups. In this respect, the performance of CHAsurf-4B was comparable to that of Curosurf®, as observed in the modified Wilhelmy balance test. Further histological analysis, as shown in Figures 4 and 5, supported these findings, providing insights into the cellular and tissue-level impacts of the treatments.

## Discussion

The criteria for an ideal artificial pulmonary surfactant, as measured by the modified Wilhelmy balance test, include: 1) rapid surface spreading, achieving an equilibrium ST of 24-27 mN/m in less than 10 seconds, 2) swift surface adsorption, attaining an equilibrium ST of 27-30 mN/m within a minute, 3) maintaining a minimal ST under 10 mN/m with only 20-30% surface compression area, 4) achieving a maximum ST of 27-30 mN/m with 100% surface compression area off, and 5) exhibiting very low surface compressibility (less than 0.03 cm/dyne at an ST of 10 mN/m) 12. Our investigation revealed that CHAsurf-4 not only met the stringent criteria for an ideal artificial pulmonary surfactant but also demonstrated superior surface activity compared to other formulations tested, marking it as a prime candidate for further development. Among the variants of CHAsurf-4, including 4A, 4B, 4C, and 4D, which correspond to increasing dosages of SP-B, CHAsurf-4B was specifically chosen for further investigation and application. This selection was based on a comprehensive analysis of critical factors. Modified Wilhelmy balance tests and in vitro experiments showed that increasing SP-B concentrations led to a marked improvement in surface tension reduction, with CHAsurf-4B demonstrating a significant enhancement in performance that aligned optimally with our study's goals. Although higher dosages of SP-B, as in CHAsurf-4C and 4D, continued to improve surfactant functionality, the incremental benefits were considered alongside the challenges of clinical application due to higher peptide concentrations. Therefore, CHAsurf-4B emerged as the preferred choice, offering an optimal blend of efficacy and feasibility for clinical translation. The effectiveness of CHAsurf-4B was subsequently confirmed in animal experiments. These findings strongly suggest that CHAsurf-4, especially CHAsurf-4B, holds significant potential as a synthetic pulmonary surfactant, poised to make a substantial impact on respiratory care.

In this research, we developed surfactants that include phospholipids, as well as both SP-B and SP-C. Specifically, our chosen surfactant, CHAsurf-4, in this research is distinguished by its Cys-Cys intradisulfide SP-B structure. The incorporation of Cys-Cys intradisulfide bonds in synthetic surfactants like CHAsurf-4 can positively impact their properties. These disulfide bonds, robust covalent connections between sulfur atoms in cysteine residues, play a pivotal role in providing structural stability to proteins, including surfactants 13. This stability, essential for maintaining the surfactant's surface activity, is achieved through the formation of these bonds. Additionally, cysteine residues, with their unique ability to form disulfide bonds, are crucial in stabilizing protein structures and maintaining their functional conformations 14.

In addition to the above, we would like to address potential concerns regarding the terminal cysteine residue in the SP-B4 structure. It is a valid consideration that the terminal Cys in SP-B4 might exhibit a tendency for intermolecular disulfide bond formation, potentially leading to dimerization or polymerization, which could affect the surfactant's function 15. While our current designs incorporate Cys-Cys intradisulfide bonds to confer structural stability to the SP-B analogs, the possibility of intermolecular binding cannot be excluded. Such interactions could inadvertently contribute to the surfactant's physical properties and behavior, particularly in a biological environment where multiple protein molecules may interact. However, in the absence of direct spectroscopic evidence, the extent to which this occurs remains speculative 16. As such, we acknowledge this as a limitation of our study and suggest it as an area for future research. Further investigations, including spectroscopic analysis, could provide insights into the behavior of these terminal Cys residues under physiological conditions. Understanding whether and how these residues participate in intermolecular binding will be crucial for refining the design of our synthetic surfactants to ensure optimal performance and stability. It's important to note that the impact of Cys-Cys intradisulfide bonds may vary based on the surfactant's composition and usage conditions. Through extensive validation, we fine-tune these aspects to optimize the synthetic surfactant's performance, particularly for treating respiratory conditions like respiratory distress syndrome in premature infants.

To evaluate our novel synthetic surfactant's effectiveness, we conducted animal experiments involving tracheostomies, ventilator-assisted lung volume measurement, and lung biopsies on New Zealand white rabbits. Notably, in the treated animal lungs, we observed a marked reduction in alveolar collapse and enhanced lung compliance, indicating that the surfactant effectively restored alveolar structural integrity and augmented lung elasticity. These changes are indicative of improved respiratory mechanics. The biopsy findings further corroborated these observations, demonstrating notable improvements in lung histology and function following the administration of our synthetic surfactant. Exhibiting promising physical properties, such as surface activity, viscosity, and biocompatibility, our synthetic pulmonary surfactant shows great potential as a therapeutic option for lung conditions characterized by surfactant deficiencies or dysfunction. In these experiments, CHAsurf-4B demonstrated performance in ST reduction comparable to Curosurf®. Despite these promising findings, our study has several limitations, including a limited selection of candidate substances, a small sample size, a primary focus on short-term effects, and an absence of a comprehensive cost-effectiveness analysis. Future research should aim to overcome these limitations, exploring long-term outcomes, considering ethical and regulatory aspects, and conducting a more thorough comparison with existing synthetic surfactants.

In conclusion, this study represents a significant advancement in the realm of synthetic surfactants, melding a deeper understanding of the role of cysteine residues and disulfide bonds with the efficacy of a synthetic SP-B analogue in pulmonary applications. Our findings not only illuminate the nuanced interplay between these structural components but also pave the way for their strategic manipulation to improve surfactant stability and efficacy. The promising results of CHAsurf-4B in animal models, mirroring the performance of Curosurf® and indicating improved lung function, contribute valuable insights into the design of new treatments for respiratory disorders.

## Figures and Tables

**Table 1:** Designs of surfactant protein B and C peptide

Name	Sequence	
SP-B1	R(Nle)LPQLVCRLVLRSS(Nle)D	
SP-B2	AcR(Nle)LPQLVCRLVLRSS(Nle)DNH2	
SP-B3	R(Nle)LPQLVCRLVLRSS(Nle)D	Head-to-tail cyclization
SP-B4	CWLSR(Nle)LPQLVCRLVLRCS(Nle)D	Cys-Cys intra disulfide
SP-B5	R(Nle)LPQLVCRLVLRCS(Nle)D	Cys-Cys inter disulfide
SP-C	CPVHLKRLLLLLLLLLLLLLLLL	

Abbreviations: SP, surfactant protein; Cys, cysteine.

**Table 2:** Composition determination of the five new synthetic surfactant candidate materials and SP-B dosage based on the selection of CHAsurf-4 among them

	DPPC (mg)	POPG (mg)	PA (mg)	SP-B (mg)	SP-C (mg)
CHAsurf-1	75	25	10	3	3
CHAsurf-2	75	25	10	3	3
CHAsurf-3	75	25	10	3	3
CHAsurf-4	75	25	10	3	3
CHAsurf-5	75	25	10	3	3
CHAsurf-4A	75	25	10	2	3
CHAsurf-4B	75	25	10	3	3
CHAsurf-4C	75	25	10	4	3
CHAsurf-4D	75	25	10	5	3

Abbreviations: DPPC, dipalmitoylphosphatidylcholine; POPG, palmitoyl-oleoyl phosphatidylglycerol; PA, palmitic acid; SP-B, surfactant protein B; SP-C, surfactant protein C.

**Table 3:** Number and mean birth weight of newborn pups in each group

	Term control	Preterm control	Preterm Curosurf®	Preterm CHAsurf-4B
N	10	11	10	10
Birth weight (gram)	64.3	35.9	36.1	36.2
